# Hybrid polymer chain discretisation for efficient and accurate implementation of hydrolytic chain scission models

**DOI:** 10.3389/fbioe.2026.1903132

**Published:** 2026-10-01

**Authors:** Oriana De Becker, Jos Vander Sloten, Liesbet Geris

**Affiliations:** 1 Biomechanics Section, Department of Mechanical Engineering, KU Leuven, Leuven, Belgium; 2 Prometheus Division of Skeletal Tissue Engineering, KU Leuven, Leuven, Belgium; 3 Biomechanics Research Unit, GIGA in silico Medicine, University of Liège, Liège, Belgium; 4 Skeletal Biology and Engineering Research Center, KU Leuven, Leuven, Belgium

**Keywords:** bioresorbable polymers, computational modelling, degradation modelling, discrete chain scission, hybrid polymer chain discretisation, hydrolytic degradation

## Abstract

Bioresorbable polymer implants offer clinical advantages over traditional bio-inert devices, but their development is complicated by geometry-dependent degradation behaviour. Computational modelling can accelerate implant development, yet mechanistic frameworks such as the Discrete Chain Scission (DCS) model are limited by high computational cost. Consequently, efficient implementation strategies are needed to enable the application of mechanistic degradation models to realistic three-dimensional (3D) implant geometries. We propose a hybrid polymer chain discretisation strategy that reduces computational cost while preserving mechanistic fidelity maintaining accurate implementation of the underlying degradation model. Simulations of random and chain-end scission demonstrate that hybrid discretisation maintains accurate predictions of molecular weight evolution and mass loss, as compared to the full model, while reducing computational cost. Sensitivity analysis confirmed robustness across discretisation thresholds. The proposed implementation strategy was applied to a proof-of-concept case study using experimental *in vitro* degradation data of P4MC, demonstrating its ability to capture all behaviours represented by the underlying DCS model, including experimentally observed molecular weight evolution and mass loss. This novel hybrid DCS approach implementation enables scalable degradation simulations, representing a key step toward applying mechanistic modelling to realistic 3D implant geometries.

## 1 Introduction

The use of bioresorbable materials for medical applications has increased over the past decades. These materials offer several advantages compared to traditional inert biomaterials, as they can eliminate the need for secondary surgery (Tayal and Khan, 2000; Ulery et al., 2011; Al-Shalawi et al., 2023), support tissue regeneration through gradual material elimination (Ferreira et al., 2017), and may even improve healing outcomes by continuously stimulating the surrounding tissues (Tayal and Khan, 2000). Among all biodegradable materials, synthetic bioresorbable polymers are often considered suitable for non-load bearing applications due to their potential for tailorability, which can enable some control over degradation rate, mechanical strength, and biological interaction. Additionally, synthetic polymers are generally associated with good reproducibility and can often be manufactured with relatively consistent bulk and surface properties, contributing to more predictable mechanical and physical properties (Tayal and Khan, 2000; Al-Shalawi et al., 2023; Song et al., 2018; Lin and Anseth, 2013; Natu et al., 2013).

Polymer biodegradation typically involves the cleavage of polymer chains into smaller fragments. For aliphatic polyesters, degradation predominantly occurs through hydrolysis, although other mechanisms, such as enzymatic or oxidative pathways, may also contribute (Laycock et al., 2017; Soares et al., 2008). As hydrolytic degradation progresses, the accumulated small oligomers and monomers diffuse out of the polymer bulk, resulting in material loss, a phenomenon referred to as erosion (Soares and Zunino, 2010). For bioresorbable polymers, these degradation products are subsequently metabolized and eliminated by the body without inducing toxic effects (Al-Shalawi et al., 2023; Bosworth and Downes, 2010). This overall process is also known as bioabsorption (Sackett and Narasimhan, 2011). Aliphatic polyesters degrade through hydrolysis when exposed to aqueous environments, such as the human body (Ferreira et al., 2017; Ratner, 2013; Chen et al., 2011; Zhang et al., 2014; Lam et al., 2009; Bartnikowski et al., 2019). Chain scission occurs primarily at the ester bonds in the polymer backbone binding the consecutive monomers together, as illustrated in Figure 1. The rate of hydrolysis - and thus the rate of degradation - is governed by water accessibility to these ester bonds (Lin and Anseth, 2013; Bartnikowski et al., 2019). As a result, hydrophobic polymers, such as poly( ε -caprolactone) (PCL) degrade relatively slowly (Elsawy et al., 2017). Multiple factors influence the specific characteristics of polymer degradation mechanisms. Aliphatic polyesters in particular have been shown to undergo bulk degradation in the biological environment of the human body (Ferreira et al., 2017; Shockley and Muliana, 2020; Versypt et al., 2013; Leroux et al., 2020; Natu et al., 2013). In this process, the degradation occurs throughout the material, rather than being limited to the surface. However, depending on the autocatalytic nature of the polymer and the design of the medical device, this bulk degradation can become heterogeneous. Autocatalysis, driven by the acidity of carboxylic acid by-products, accelerates hydrolytic chain scission within the polymer’s core compared to its surface (Bosworth and Downes, 2010; Chen et al., 2011; Versypt et al., 2013; Heljak et al., 2014). Device geometry plays a key role in the degradation process, affecting not only the diffusion of degradation by-products (oligomers and monomers) but also the spatial distribution of mechanical property changes and mass loss (Bartnikowski et al., 2019). These geometric effects highlight the importance of incorporating degradation behaviour into the medical device design phase (Vieira et al., 2023; Versypt et al., 2013).

**FIGURE 1:**
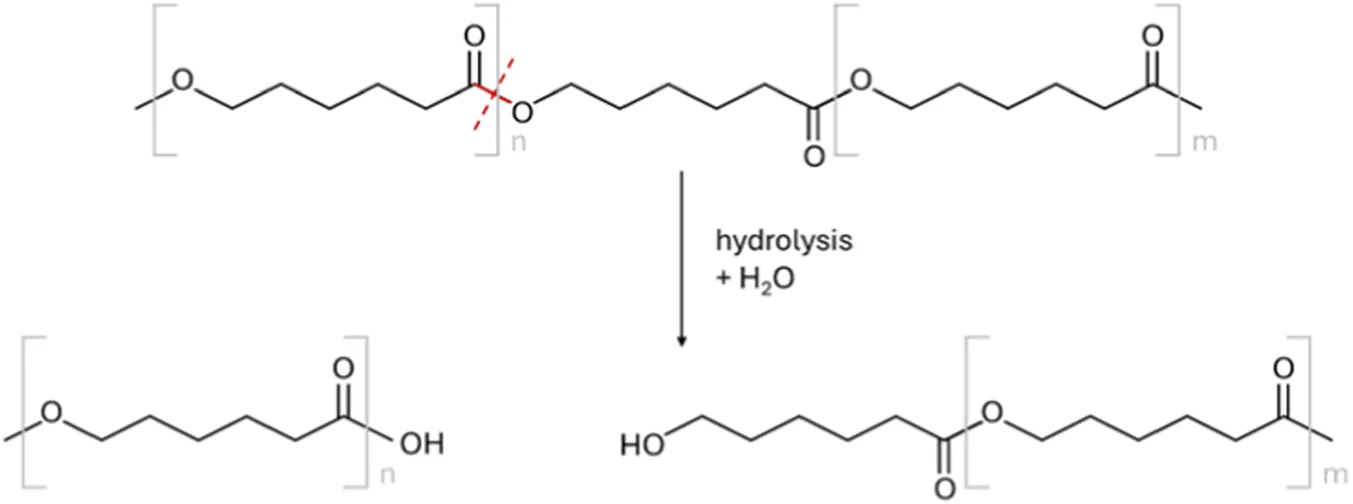
Hydrolysis of a poly( ε -caprolactone) (PCL) polymer chain.

To this end, computational and mathematical models have increasingly been employed to support the design and optimization of biodegradable medical devices. These models reduce the need for extensive experimental testing and allow for rapid exploration of various design alternatives (Versypt et al., 2013; Pemmada et al., 2024; Cabrera et al., 2017). In addition, they offer valuable insights into the underlying degradation mechanisms and their interdependencies (Versypt et al., 2013). Several mathematical models capturing hydrolytic degradation have been reported in the literature (Wang et al., 2008; Han and Pan, 2009; Heljak et al., 2014; Han, 2014; Gleadall et al., 2014; Shine et al., 2017; Sevim and Pan, 2018; Vieira et al., 2023; Batoni et al., 2023; Antheunis et al., 2009; Soares and Zunino, 2010; Pan and Brassart, 2023; Chen et al., 2011; Zhang et al., 2014; Celestine et al., 2020; Su et al., 2022; Rothstein et al., 2009; Shockley and Muliana, 2020). These models can be categorized into phenomenological models and mechanistic models. The latter are particularly powerful, as they are based on the physical and chemical processes governing polymer degradation, allowing for better extrapolation beyond experimental conditions (Versypt et al., 2013; Geris et al., 2022). A widely adopted semi-mechanistic approach is the bi-modal polymer chain simplification, originally introduced by Wang et al. (2008) (Wang et al., 2008). This framework models the polymer as consisting of short and long chain populations and has successfully been applied by multiple research groups (Heljak et al., 2014; Han and Pan, 2009; Han, 2014; Gleadall et al., 2014; Shine et al., 2017; Sevim and Pan, 2018; Vieira et al., 2023; Batoni et al., 2023). However, Gleadall et al. (2014) pinpointed a critical limitation: the bi-modal assumption cannot distinguish between chain-end and random chain scission (illustrated in Figure 2A) along the polymer backbone, which may lead to inaccuracies in predicting degradation behaviour (Gleadall et al., 2014). An alternative to this bi-modal assumption involves representing the full polymer dispersity, considering chains of all lengths x ϵ (1,N) , where N denotes the maximum number of repeating monomers in the polymer chain. Within this framework, chain scission is described using the Discrete Chain Scission (DCS) approach. The conceptual foundation of this approach dates back to Montroll and Simha, (1940), and was further developed by Batycky et al. (1997) (Batycky et al., 1997), who formulated a discrete kinetic model governed by a coupled system of linear Ordinary Differential Equations (ODEs). Antheunis et al. (2009) demonstrated the applicability of this framework to semi-crystalline polymers and incorporated autocatalytic effects (Antheunis et al., 2009). More recently, Pan and Brassart (2023) embedded this DCS modelling approach within a reaction-diffusion framework to account for the diffusion of small, soluble polymer chains out of the device, thereby capturing mass loss during degradation (Pan and Brassart, 2023).

**FIGURE 2:**
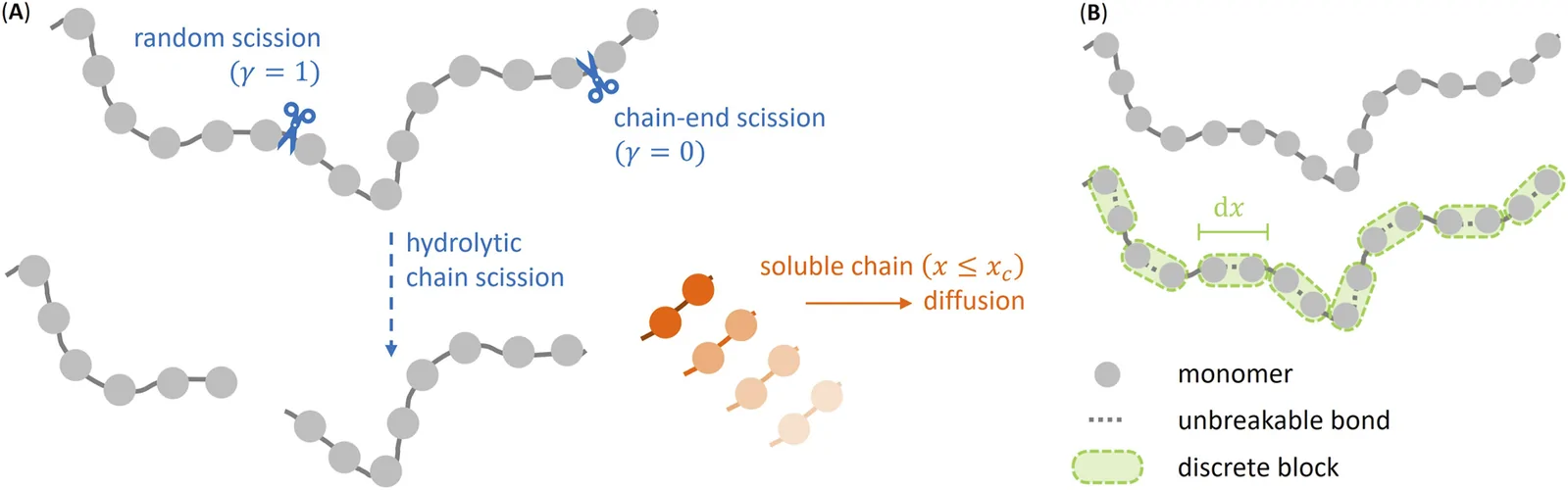
**(A)** Schematic illustration of random scission, chain-end scission, and diffusion of short, soluble chains. Polymer chains are represented as sequences of grey circles (monomers) and short, soluble chains are shown in orange. **(B)** Conceptual illustration of uniform polymer chain discretisation, where dx monomers are grouped into discrete blocks (shown in green). γ = weighing parameter balancing random and chain-end scission; $x_{c}$ = solubility chain length threshold below which chains are soluble; dx = discretisation step, i.e., the number of monomers grouped into a single discrete block.

Despite the detailed mechanistic insights offered by the DCS modelling approach, its computational demand poses a significant challenge, particularly when applied to realistic device geometries. A key factor governing this computational cost is the number of repeating monomers N in the longest polymer chain considered. A potential strategy to mitigate this cost is to discretise the polymer chain by grouping multiple monomers into a single block and thus reducing the number of discretised blocks (Figure 2B) (Soares and Zunino, 2010). Unlike conventional coarse-graining approaches used in polymer modelling, such as bead-spring or rod-chain representations, the purpose of this discretisation is not to introduce a physically meaningful mesoscale description of the polymer microstructure. Rather, it serves as a numerical implementation strategy intended to reduce the computational cost associated with the DCS framework. To date, little attention has been paid to how the level of polymer chain discretisation affects the balance between model accuracy and computational feasibility, a trade-off crucial for practical applications. This work directly addresses this gap by investigating the impact of polymer chain discretisation in modelling both chain-end and random chain scission. To isolate the effect of chain discretisation, we evaluated multiple discretisation levels using the DCS modelling strategy in its stand-alone form, without incorporating it within a broader reaction-diffusion framework. Building on these insights, we propose a hybrid discretisation implementation strategy for the DCS model that balances computational efficiency with predictive accuracy, enabling scalable simulation of hydrolytic polymer degradation. Given the limited availability of suitable experimental datasets, the case study is intended to demonstrate the ability of the implementation strategy to capture all behaviours represented by the underlying DCS model, including experimentally observed molecular weight evolution and mass loss.

## 2 Materials and methods

### 2.1 Discrete Chain Scission (DCS) model description

The DCS model has been described in detail in the literature (Soares and Zunino, 2010), a brief overview of its key principles is provided here.

The model describes the time evolution of weight concentration of polymer chains of length x , denoted by $w_{x}\left(t\right)$ (Gavrilov and Monteiro, 2015), where $w_{x}$ represents the weight fraction of chains of length x . When considered in its stand-alone form, neglecting the diffusion of short, soluble chains, the governing equations for all chain lengths x ϵ (1,N) reduce to a system of Ordinary Differential Equation (ODE) (Equation 1). Since diffusion is not explicitly accounted for, the weight fractions of chains with lengths below the solubility threshold $x_{c}$ are set to zero $\left(w_{x\leq x_{c}}\left(t\right)=0\right)$ to represent mass loss.

$$\frac{\partial w_{x}}{\partial t}=R_{x}=\nu_{x}\;\dot{\xi }$$ (1)

Here, $R_{x}\left(t\right)$ represents the net production rate of chains of length x due to chain scission. The term $\dot{\xi }\left(t\right)$ defines the rate of chain scission events per unit volume and per time unit, while $\nu_{x}\left(t\right)$ is a dimensionless coefficient indicating how many chains of length x are produced (or lost, if negative) per scission event. This coefficient enables the model to represent different chain scission mechanisms, such as random and chain-end scission.

The model assumes that chains of length x can be either degraded *via* hydrolysis or produced as degradation products of longer polymer chains (Pan and Brassart, 2023). This is described by:

$$\nu_{x}=-\overset{x-1}{\underset{y=1}{\sum }}k_{x,y}w_{x}+\overset{N}{\underset{y=x+1}{\sum }}\left(k_{y,x}+k_{y,y-x}\right)w_{y}\;.$$ (2)

In this expression, $k_{x,y}$ denotes the probability that a chain of length x undergoes cleavage at the $y^{th}$ bond, normalised by the total number of chain scission events per unit volume. The variables $k_{y,x}$ and $k_{y,y-x}$ account for the formation of chains of length x as products of longer chains breaking at different positions (the $x^{th}$ bond and ${\left(y-x\right)}^{th}$ bond respectively).

To ensure physical consistency, the coefficients $\nu_{x}$ must satisfy two constraints. First, each hydrolytic chain scission event must produce exactly one new polymer chain:

$$\overset{N}{\underset{x=1}{\sum }}\nu_{x}=1\;.$$ (3)

Second, since hydrolysis is a bond cleavage process, it conserves the total number of monomers in the system:

$$\overset{N}{\underset{x=1}{\sum }}x\nu_{x}=0\;.$$ (4)

From Equation 3, a corresponding constraint on the coefficients $k_{x,y}$ can be derived (Equation 5), ensuring that each scission event produces exactly one additional polymer chain. The full derivation of this constraint can be found in the Supplementary Material.

$$\overset{N}{\underset{x=2}{\sum }}w_{x}\overset{x-1}{\underset{y=1}{\sum }}k_{x,y}=1$$ (5)

### 2.2 Chain scission mechanisms

Given this model description, Pan et al. (2023) introduced three different chain scission mechanisms that can be incorporated into Equation 2: random chain scission, chain-end scission, and a combined mechanism. We implemented both random and chain-end scission, and further extended this framework to generalise the definition of chain-end scission. Additionally, we propose an alternative and more flexible approach for implementing combined chain scission within the DCS framework.

#### 2.2.1 Random chain scission

Firstly, random chain scission is characterised by equal probability of hydrolytic scission regardless of the position of the bond along the polymer chain. In this case, all coefficients $k_{x,y}$ are identical (i.e., $k_{x,y}=k$ ). Using Equation 5, the value of k can be derived as:

$$k=\frac{1}{C_{b}}\;.$$ (6)

Where $C_{b}$ is the total concentration of bonds, $C_{b}\;=\;\sum_{x=1}^{N}\;w_{x}\;\left(x-1\right)$ . This relationship highlights an increasing probability of hydrolysis for individual bonds as the total concentration of bonds decreases during degradation.

#### 2.2.2 Chain-end scission

Secondly, chain-end scission is defined as the mechanism in which only the outermost bonds of a polymer chain (i.e., the first and last bonds) are susceptible to hydrolysis. The scission probability $k_{x,y}$ is therefore defined as:

$$k_{x,y}=\left\{\begin{array}{cc} k & if\;y=1\;or\;y=x-1\\ 0 & otherwise \end{array}\right.\;.$$ (7)

Following Equation 5, the corresponding value for k is:

$$k=\frac{1}{2W_{p}-w_{2}-2w_{1}}\;.$$ (8)

Here, $W_{p}$ represents the total concentration of polymer chains, defined as $W_{p}=\sum_{x=1}^{N}w_{x}$ , and $w_{1}$ and $w_{2}$ represent the concentration of chains of length one and two, respectively.

#### 2.2.3 Extended chain-end scission

Thirdly, we propose an extension to the chain-end scission mechanism in which not only the single outermost bond, but the outermost ‘ a ’ bonds on each side of the polymer chain are susceptible to hydrolysis. The scission probability function $k_{x,y}$ becomes:

$$k_{x,y}=\left\{\begin{array}{cc} k & if\;y=1,\ldots ,a\;or\;y=x-1,\ldots ,x-a\\ 0 & otherwise \end{array}\right.\;.$$ (9)

Applying the same derivation approach as for Equation 8, the value of k is found to be:

$$k=\frac{1}{2aW_{p}-\sum_{x=1}^{2a}\left(2a-\left(x-1\right)\right)w_{x}}\;.$$ (10)

For a=1 , Equation 10 reduces to the expression in Equation 8, thereby recovering the original chain-end scission formulation.

#### 2.2.4 Combined mechanism

Lastly, Pan et al. (2023) introduced a separate definition for the $k_{x,y}$ values to model combined scission mechanisms, where chain-end and random chain scission are balanced through a weighing parameter β . Although this framework could be generalised to the extended chain-end mechanism, it is not compatible with the proposed hybrid discretisation strategy. To address this, an alternative approach was adopted for the combined chain scission mechanism (Equation 11).

$$\frac{\partial w_{x}}{\partial t}=-\nabla \;\;J_{x}+\nu_{x,end}\;{\dot{\xi }}_{\mathrm{end}}\;\left(1-\gamma \right)+\nu_{x,random}\;{\dot{\xi }}_{\mathrm{random}}\;\gamma$$ (11)

In this formulation, γ serves the same conceptual role as β by weighing the contribution of chain-end *versus* random scission. However, due to the difference in implementation, γ does not correspond numerically to β , even when representing similar behaviour.

The full derivations of all equations referred to in this section are provided in the Supplementary Material.

### 2.3 Complementary kinetic model

As illustrated in Equation 11, the net production rate of polymer chains $R_{x}\left(t\right)$ depends not only on $\nu_{x}\left(t\right)$ , the fragmentation term, but also on the chain scission rate $\dot{\xi }\left(t\right)$ . In the present work, the scission rate is governed by a semi-empirical kinetic law inspired by the work of Wang et al. (2008), who employed the bi-modal approximation to describe hydrolytic degradation (Wang et al., 2008). Although originally applied in simplified models, this kinetic formulation can also be integrated into more advanced frameworks such as the DCS model (Pan and Brassart, 2023).

The rate of hydrolytic degradation is described by two contributions: a first-order rate constant $k_{1}$ , and an autocatalytic term involving a second constant $k_{2}$ , which accounts for the autocatalytic nature of the process:

$$\dot{\xi }\left(X,t\right)=\left(k_{1}+k_{2}{\left(K_{a}w_{1}\right)}^{m/2}\right)C_{b}^{n}\;.$$ (12)

Here, $K_{a}$ is the acid dissociation equilibrium constant. The exponents m and n are model parameters, often set to unity for simplicity (Pan and Brassart, 2023). However, in the present model formulation, mass loss is implicitly incorporated by setting $w_{x\leq x_{c}}=0$ . As a result, the autocatalytic contribution disappears, and the kinetic model reduces to a purely first order form:

$$\dot{\xi }\left(X,t\right)=k_{1}C_{b}^{n}\;.$$ (13)

As the primary objective of this work is to evaluate the proposed hybrid discretisation implementation rather than to develop a polymer-specific degradation model, the simplified first-order kinetics were considered sufficient for the present analysis. The proposed discretisation implementation strategy is independent of the underlying kinetic formulation and can therefore also be applied within more advanced DCS-based degradation frameworks that explicitly account for autocatalysis (Pan and Brassart, 2023).

### 2.4 Polymer chain discretisation

While the DCS framework, particularly with the proposed generalisations, provides a robust mechanistic description of hydrolytic polymer degradation, its computational cost strongly depends on the maximum polymer chain length N considered in the simulations. Moreover, the computational cost increases more than linearly with increasing N , highlighting the need for strategies that balance model accuracy with computational feasibility.

Building on the work of this study adopts a polymer chain discretisation strategy Soares and Zunino (2010). In their work, Soares et al. demonstrated that, for random scission, discretisation did not significantly affect the predicted evolution of molecular weight. However, their analysis did not consider chain-end scission mechanisms, nor did it investigate the impact of discretisation on mass loss. These missing aspects are relevant since both are crucial for accurately modelling hydrolytic degradation of bioresorbable aliphatic polyesters.

In the proposed uniform discretisation strategy, monomers are grouped into blocks of size dx , such that bonds within a block are assumed unbreakable while only the bonds between consecutive blocks remain susceptible to hydrolysis. This concept is schematically illustrated in Figure 2B. This uniform discretisation definition leads to two important implications.

First, for random scission, discretisation artificially increases the likelihood of creating soluble fragments (defined by Equation 14). Moreover, this artificial increase becomes more pronounced with decreasing polymer chain length x as illustrated in Figure 3. Therefore, a uniform discretisation across all chain lengths in not suitable, since it would lead to discretisation-dependent mass loss predictions. To address this issue, we propose a hybrid discretisation strategy in which all chains shorter than a predefined critical chain threshold the hybrid discretisation threshold are modelled in full detail, while only chains longer than this threshold are discretised. This approach, illustrated in Figure 4, preserves accuracy where mass loss is most sensitive to discretisation while substantially reducing the computational cost associated with very long chains.

$$P_{\mathrm{soluble}}=\frac{2x_{c}/dx}{x/dx-1}$$ (14)

**FIGURE 3:**
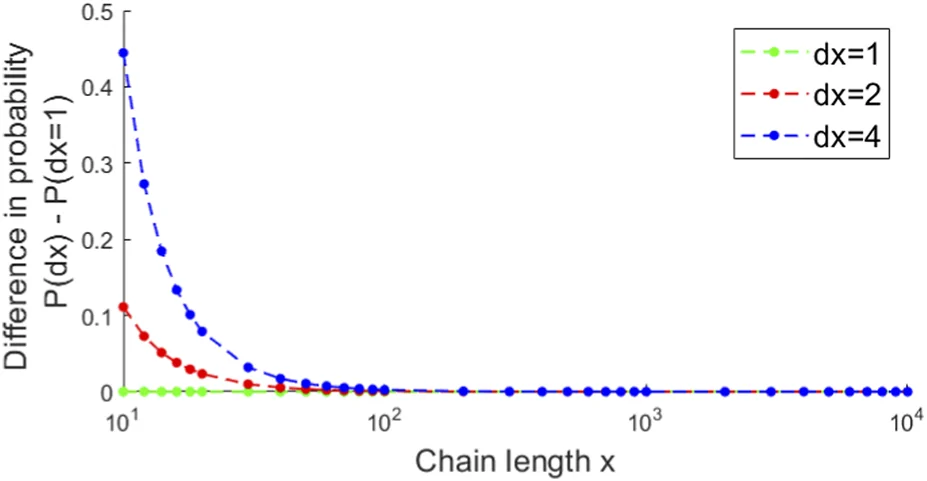
Difference in probability of creating soluble chains following a random chain scission mechanism as a function of polymer chain length x . Results are shown for three different discretisation levels dx=1,2,4 (green, red, blue respectively).

**FIGURE 4:**
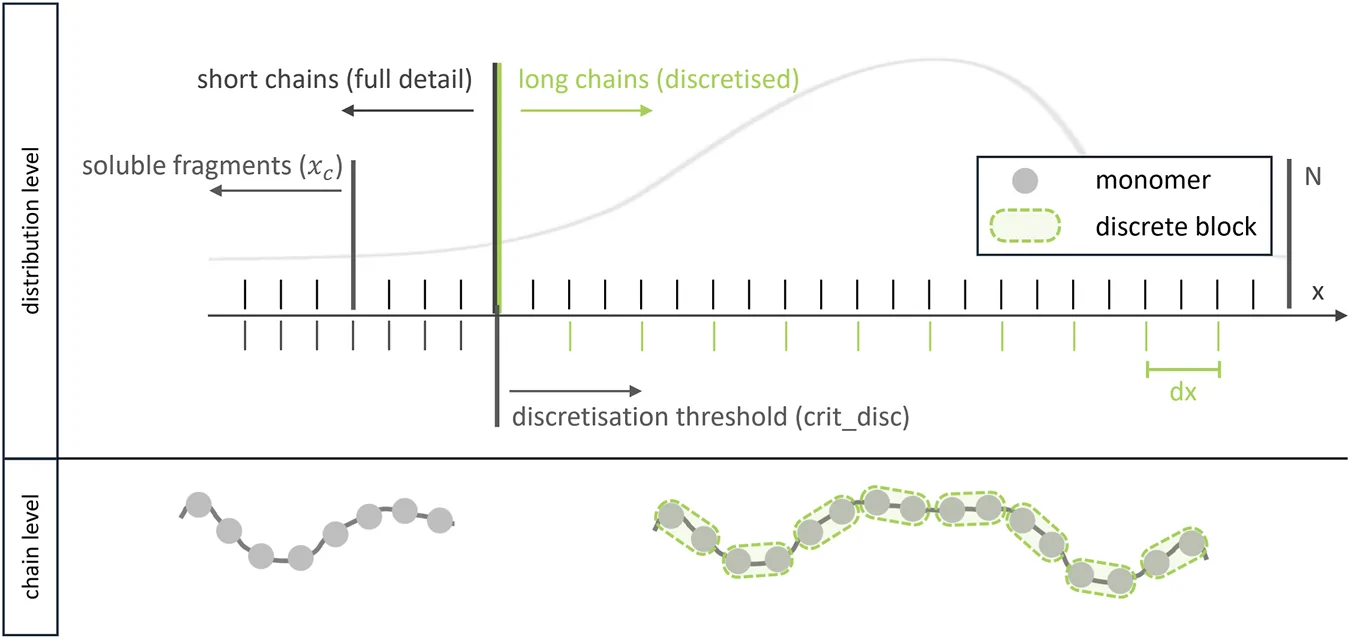
Schematic illustration of the hybrid polymer chain discretisation strategy. x = number of repeating monomers in a polymer chain; N = maximum number of repeating monomers (i.e. longest chain considered); $x_{c}$ = critical chain length below which chains are soluble; crit_disc = hybrid discretisation threshold above which chains are discretised; dx = discretisation step, i.e. the number of monomers grouped into a single discrete block.

Here, $x_{c}$ represents the threshold for soluble monomers and dx the discretisation level.

Second, Equation 15 represents the expected mass loss per chain scission event. It is computed as the average mass of all possible soluble fragments, obtained by summing the mass contributions of fragments up to the solubility threshold $x_{c}$ , discretised in steps of dx , and dividing by the total number of discrete fragment sizes considered. This expression illustrates how discretisation alters the expected mass removed per scission event in case of chain-end scission. This effect is specific to the chain-end scission mechanism and does not apply to random scission, highlighting the need for the previously introduced partitioned implementation that treats both mechanisms consistently. To account for this discretisation-dependent chain-end scission rate, the chain-end scission rate is explicitly compensated according to Equation 16 within the discretised chains.

$$E\left[ML\right]=\frac{\sum_{x=1}^{x_{c}/dx}xdx}{x_{c}/dx}$$ (15)

$${\dot{\xi }}_{\mathrm{end,compensated}}=\frac{x_{c}/dx\sum_{x=1}^{x_{c}}x}{x_{c}\sum_{x=1}^{x_{c}/dx}xdx}{\dot{\xi }}_{\mathrm{end}}$$ (16)

The proposed hybrid discretisation strategy, combined with a correction for discretisation-dependent chain-end scission rates, achieves computational efficiency without compromising mechanistic fidelity of the different degradation pathways, a hypothesis that will be verified in the following section.

### 2.5 Model credibility analysis

To evaluate the stated hypothesis and establish the credibility of the DCS modelling strategy with hybrid polymer chain discretisation, two types of analyses were performed. First, the model was run at three discretisation levels, corresponding to block sizes of one (reference), two, and four repeating monomer units. These levels allow assessment of how grouping the monomers into larger blocks affects model predictions. This analysis was conducted for both random (γ=1) and chain-end scission (γ=0) , and the resulting evolutions of molecular weight distribution, weight-average molecular weight, and mass loss were compared. The weight- and number-average molecular weight (g/mol) and mass loss (%) were computed from the molar mass distribution using Equations 17, 18, 19, respectively.

$$M_{w}=M_{1}\frac{\sum_{x=1}^{N}n_{x}x^{2}}{\sum_{x=1}^{N}n_{x}x}=M_{1}\frac{\sum_{x=1}^{N}w_{x}x}{\sum_{x=1}^{N}w_{x}}$$ (17)

$$M_{n}=M_{1}\frac{\sum_{x=1}^{N}n_{x}x}{\sum_{x=1}^{N}n_{x}}$$ (18)

$$ML=1-\frac{\sum_{x=1}^{N}w_{x}x}{\sum_{x=1}^{N}w_{x,0}x}$$ (19)

In these equations, $n_{x}$ and $w_{x}$ denote the number and weight fractions ( $w_{x}=Mn_{x}$ ,with $M=xM_{1}$ ), respectively, of chains containing x discretised units (Gavrilov and Monteiro, 2015). The variable x represents the number of monomers in the chain and $M_{1}$ is the molar mass of one monomer (g/mol) . The initial weight fraction distribution $w_{x,0}$ was defined as an arbitrary log normal with shape parameters μ=500 and σ=0.2 and a maximum degree of polymerization of N=2000 . The shape parameters were selected to generate a representative unimodal molecular weight distribution, while the maximum degree of polymerization was chosen to ensure a physically relevant chain length range consistent with the subsequent case study. The solubility threshold $x_{c}$ , defining the upper bound for soluble chains, and the hybrid discretisation threshold were set to 4 and 400, respectively. The solubility threshold was selected to remain consistent with the subsequent case study, whereas the hybrid discretisation threshold was determined based on the sensitivity analysis described below.

Second, the model’s sensitivity to the hybrid discretisation threshold that determines whether a chain is modelled in full detail or discretised was evaluated for the random scission mechanism. This analysis looks at how the threshold selection influences prediction accuracy and computational cost, ensuring the hybrid approach remains robust across reasonable choices of said threshold.

### 2.6 Case study: *In vitro* degradation of P4MC

Lastly, we performed a case study to assess both the credibility of the model under a combined scission mechanism and the application potential of the DCS modelling strategy. The case study is based on experimental data reported by Antheunis et al. (2009) (Antheunis et al., 2009). They studied the degradation of several polymers, including the viscous liquid poly(4-methylcaprolactone) (P4MC), in Phosphate Buffered Saline (PBS) buffer at 37°C for just over 400 days. P4MC was chosen because its viscous liquid form implies that the assumption of instantaneous diffusion is valid, which allows the application of this spatially independent DCS formulation (Pan and Brassart, 2023).

A Genetic Algorithm (GA) was employed to determine the model parameters that best fit the experimental data (Mukherjee et al., 2025). The GA optimisation was performed using a single run with a population size of 50 individuals and was terminated when either a maximum of 30 generations was reached or when no improvement in the best objective-function value was observed for 10 consecutive generations. The optimisation was performed in two steps: first, the weighing parameter γ was optimised based on the molecular weight - mass loss map, and second, the kinetic parameter $k_{1}$ was optimised based on the time evolution of both the weight-average molecular weight and mass loss. The initial weight fraction distribution was assumed to follow a log normal distribution with shape parameters μ=102 and σ=1.21 , and a maximum degree of polymerization of N=2000 . These shape parameters were adopted from Pan and Brassart (2023), who calibrated them to reproduce the experimentally observed initial molecular weight (Pan and Brassart, 2023). The maximum degree of polymerization was chosen to ensure consistency with the observed initial molecular weight. The solubility threshold $x_{c}$ and the hybrid discretisation threshold were kept at 4 and 400, respectively. The hybrid discretisation threshold was selected based on the sensitivity study, while the solubility threshold was derived from experimental data reported by Antheunis et al. (2009). Using High-Performance Liquid Chromatography-Mass Spectrometry, they identified the longest oligomer chains present in the degradation medium, providing an experimental estimate of the critical chain length at which polymer fragments become water-soluble (Antheunis et al., 2009).

This case study is not intended as a formal validation of the DCS framework, as it is based on a single experimental dataset. Instead, its purpose is to assess whether the proposed DCS framework, implemented with a hybrid discretised polymer chain, yields a physically meaningful description of hydrolytic degradation. In this context, the case study is considered successful if the model demonstrates its ability to reproduce both the time evolution of molecular weight and mass loss within a single modelling framework and parameter set. Moreover, this consistency should be maintained across different levels of discretisation.

## 3 Results

### 3.1 Effect of hybrid polymer chain discretisation on model predictions and computational cost

We evaluated the effect of hybrid polymer chain discretisation on both computational cost and model outputs. Figure 5 shows the computational cost of the DCS model for three discretisation levels corresponding to block sizes of one (reference), two, and four repeating monomer units using the hybrid discretisation strategy. Comparing the reference cases reveals that the updated implementation required to enable the hybrid discretisation framework induces an initial increase in computational cost by almost a factor of two. However, once discretisation is applied, the updated model enables a substantial reduction in computational cost, bringing it far below the reference level of the non-discretised implementation.

**FIGURE 5:**
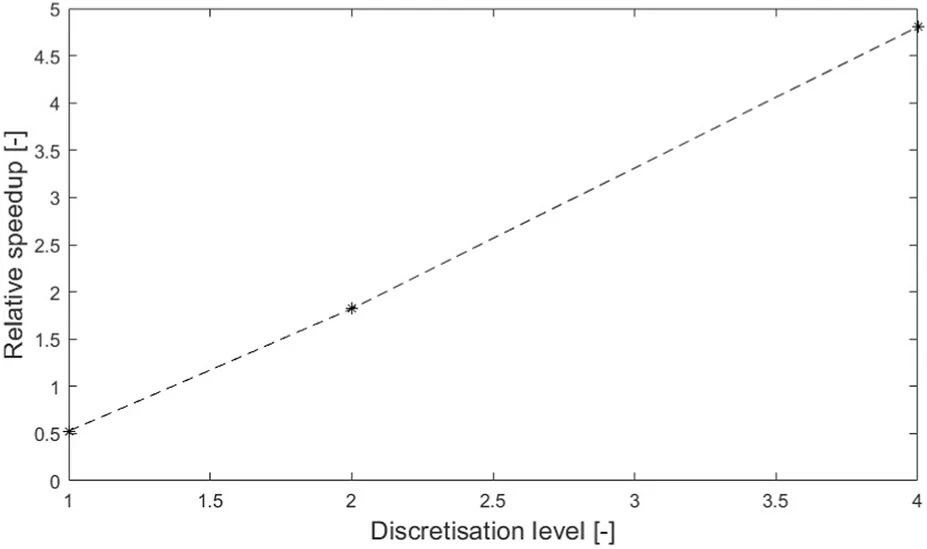
Relative speedup of the Discrete Chain Scission (DCS) model for three discretisation levels (block sizes of one, two, and four repeating monomer units), calculated with respect to the non-discretised reference implementation. Values greater than one indicate improved computational performance.

To quantify the influence of discretisation on model predictions, the random chain scission mechanism was simulated using the same three discretisation levels (dx=1,2,4) , with dx=1 serving as the reference. The results are presented in Figures 6A,B. Figure 6A shows the evolution of the molar mass distribution over normalised time for the different discretisation levels. Time is normalized with respect to the point at which the weight-average molecular weight reaches half of its initial value. The molar mass distribution is represented as a differential log molecular weight distribution x(M) derived from the weight distribution according to Equation 20 (with $log_{10}e=0.443429448$ ) (Gavrilov and Monteiro, 2015). Figure 6B presents the corresponding evolution of the weight-average molecular weight and mass loss.

$$x\left(M\right)=\frac{M}{log_{10}e}w\left(M\right)$$ (20)

**FIGURE 6:**
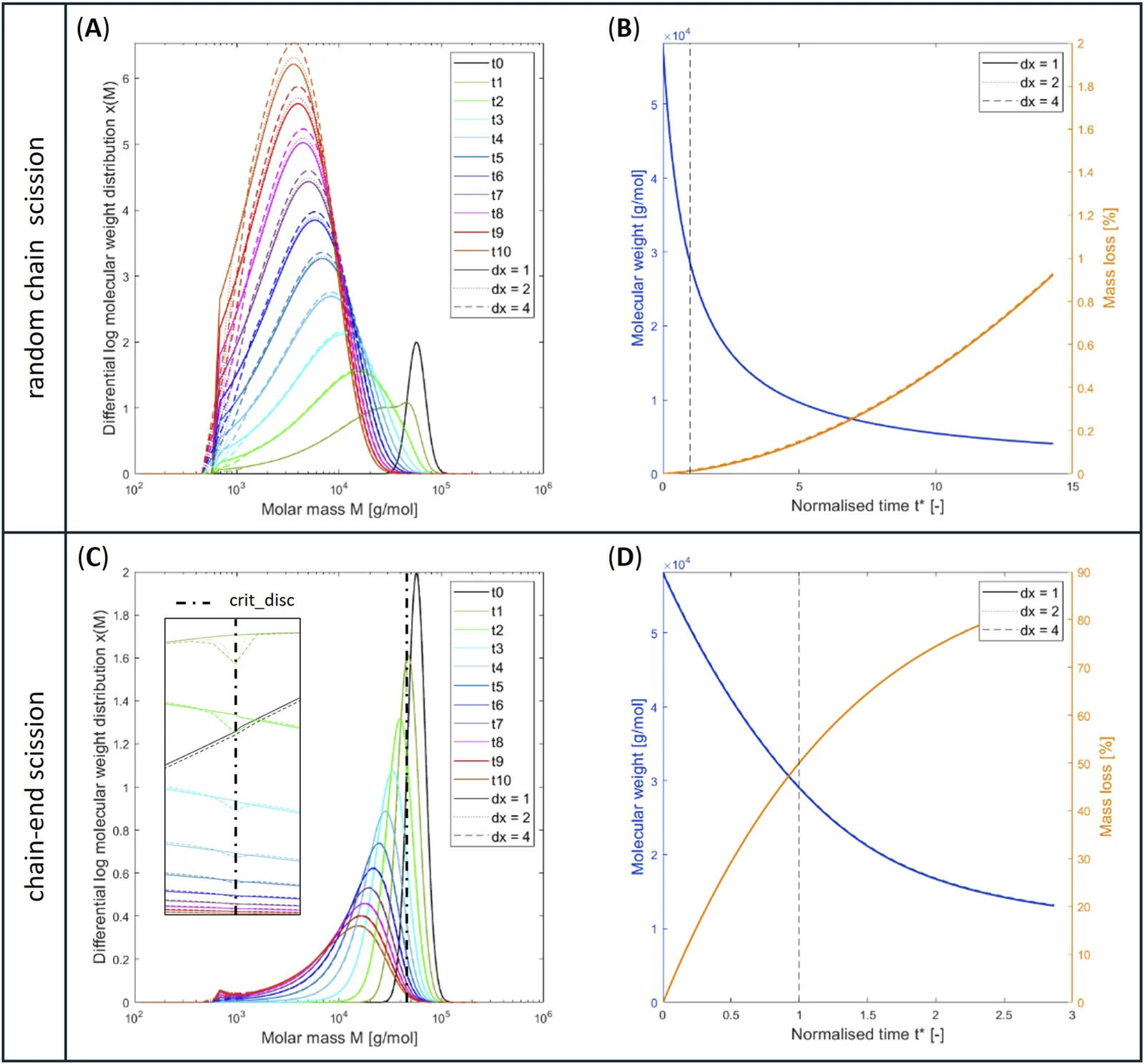
Model outputs at different time points (t) from simulations for three discretisation levels (block sizes (dx) of one, two, and four repeating monomer units; full, dotted, and dashed lines, respectively). If only one curve is visible, the results from different discretisation schemes overlap. **(A)** Evolution of polymer molar mass distribution following random chain scission. **(B)** Evolution of weight-average molecular weight (blue) and mass loss (orange) following random chain scission. **(C)** Evolution of polymer molar mass distribution following chain-end scission. **(D)** Evolution of weight-average molecular weight (blue) and mass loss (orange) following chain-end scission.

A similar analysis was performed for the chain-end scission mechanism using the same discretisation levels, with results shown in Figures 6C,D. Figure 6C shows the evolution of the molar mass distribution over normalised time. A zoomed-in view around the hybrid discretisation threshold (crit_disc) is included, highlighting a local transition in the distribution at this threshold. However, this feature does not seem to propagate or amplify over time. Figure 6D presents the corresponding evolution of the weight-average molecular weight and mass loss.

Overall, these results indicate that the hybrid discretisation strategy has a limited impact on model predictions, particularly with respect to the evolution of weight-average molecular weight and mass loss.

Lastly, Figure 7 presents the sensitivity of the chain length hybrid discretisation threshold used in the hybrid polymer chain discretisation. Figure 7A shows the resulting weight-average molecular weight and mass loss, while Figure 7B displays the corresponding computational cost. Lower threshold values lead to a larger portion of chains being discretised, thereby reducing computational cost. However, for relatively short chains, discretisation introduces an artificial increase in mass loss (Figure 3), as the likelihood of producing soluble fragments is dependent on the polymer chain discretisation.

**FIGURE 7:**
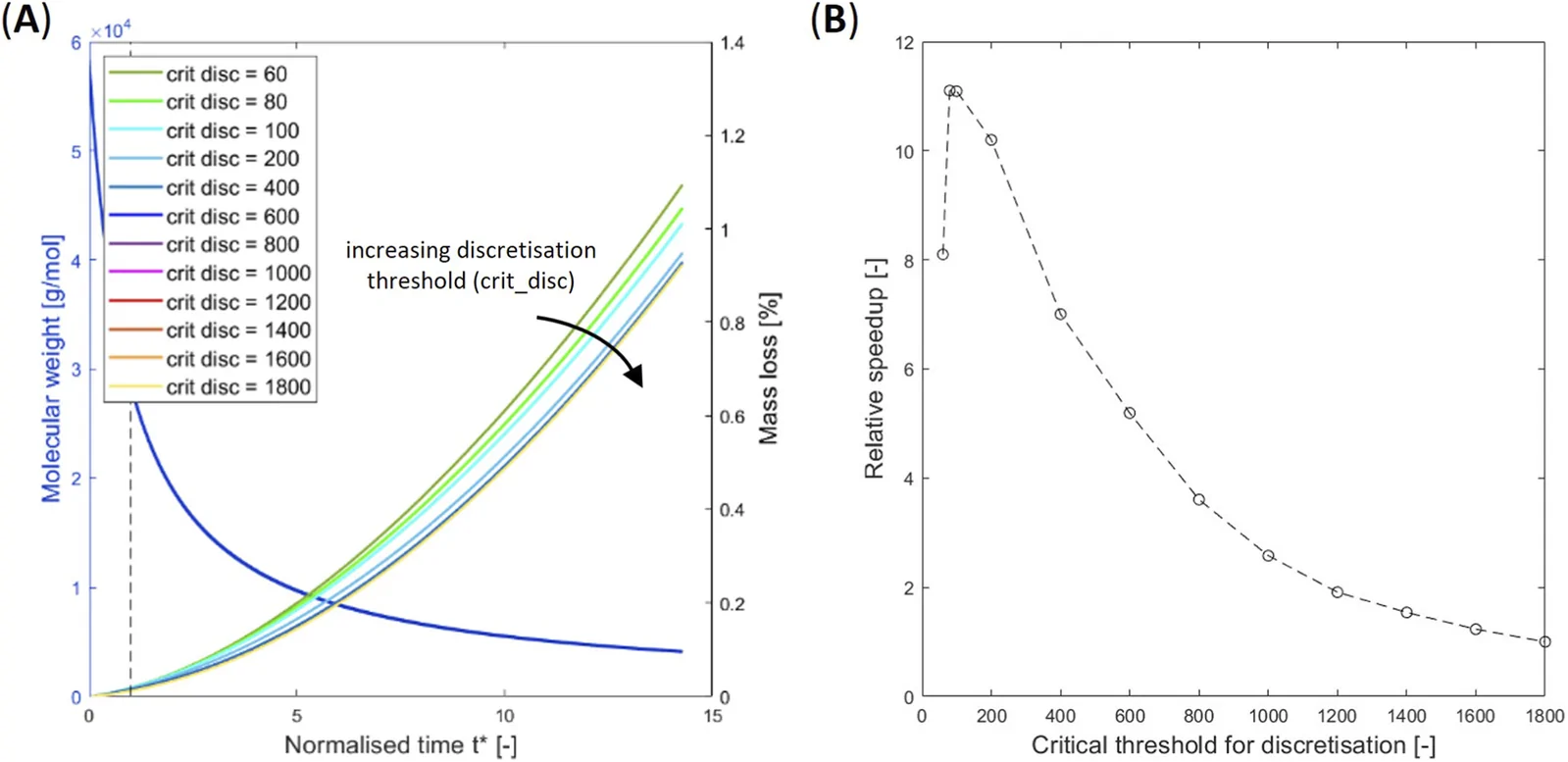
Sensitivity analysis for the critical chain length hybrid discretisation threshold (crit_disc) for polymer chain discretisation. **(A)** Evolution of weight-average molecular weight and mass loss following random chain scission from simulations run with different hybrid discretisation thresholds (represented by different colors on the plot and in the legend). Only five curves are visually distinguishable, the remaining curves, corresponding to higher thresholds, overlap with the lowest curve. **(B)** Relative speedup as a function of the hybrid discretisation threshold, calculated with respect to the computational cost obtained for a hybrid discretisation threshold of 1800.

### 3.2 Case study: et al. Degradation of P4MC

*In vitro* experimental data from Antheunis et al. (2009) was used to calibrate the DCS model through a two-step GA-based parameter optimisation (Antheunis et al., 2009). The weighing parameter γ was first optimised using the weight-average molecular weight - mass loss map (Figure 8A). In this figure, the circular symbols (‘ o ’) represent the experimental data and the solid line shows the model prediction for γ=0,9298 . In the second step, the kinetic parameter $k_{1}$ was optimised, yielding a value of $6.1240\;10^{-5}\;day^{-1}$ . The resulting evolution of weight- and number-average molecular weights and mass loss are shown in Figures 8B,C. These results show that, with optimised parameters, the model framework is able to reproduce experimental trends in molecular weight evolution and mass loss, supporting the suitability of the discretised DCS formulation for capturing the degradation behaviour under the studied conditions.

**FIGURE 8:**
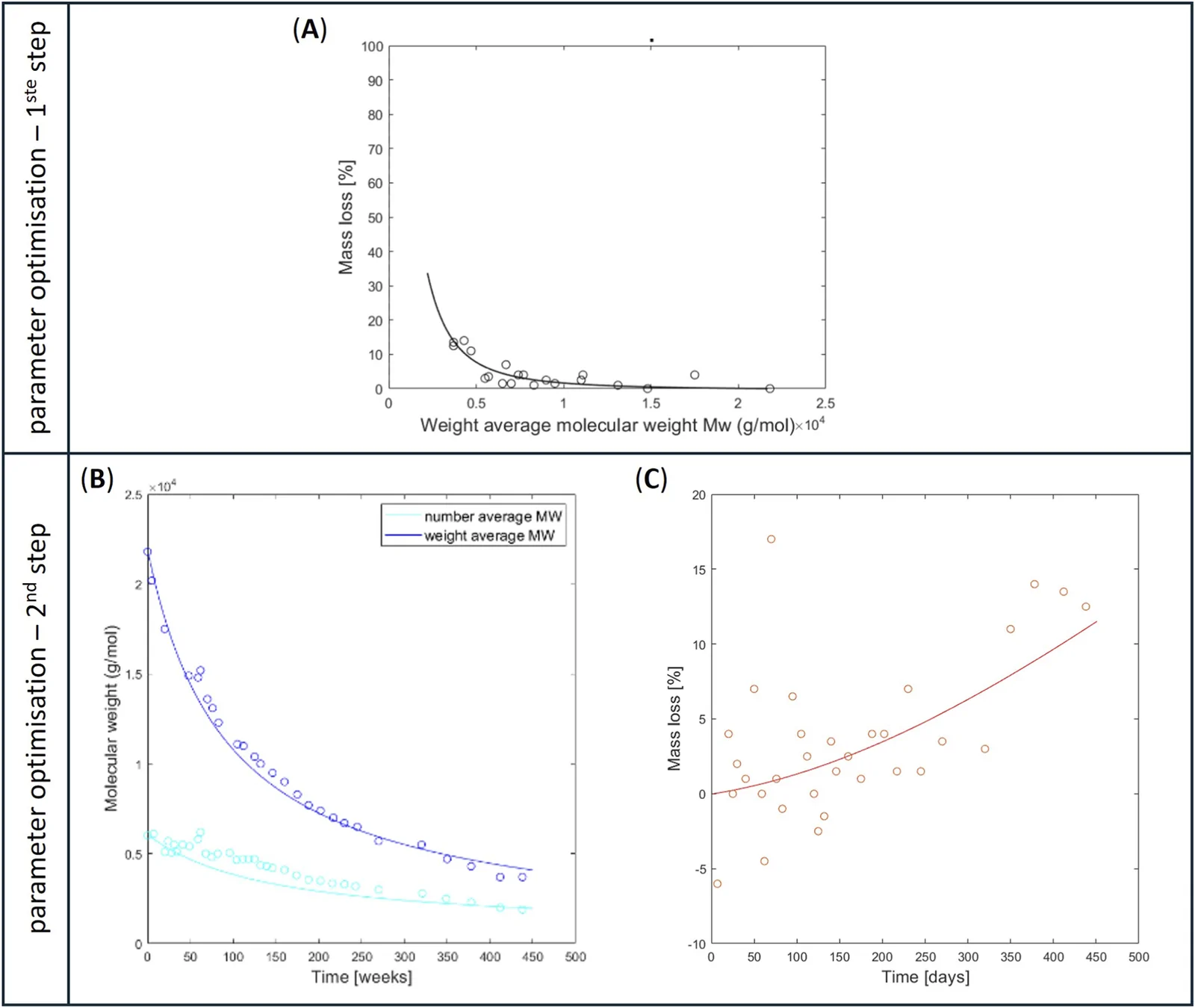
The symbols (’o’) represent the experimental data reported by Antheunis et al. (2009), and the solid line shows the GA-optimised model predictions obtained using the optimised value of weighing parameter γ and kinetic parameter $k_{1}$ (Antheunis et al., 2009). **(A)** Molecular weight–mass loss map. Evolution of **(B)** weight-average (dark blue, top) and number-average (light blue, bottom) molecular weight and **(C)** mass loss during degradation.

## 4 Discussion

The results presented in this study demonstrate that a hybrid polymer chain discretisation implementation strategy can be effectively combined with the DCS framework to capture hydrolytic chain scission while substantially reducing computational cost. As shown in Figure 6, introducing hybrid discretisation does not significantly alter the evolution of molecular weight metrics or mass loss, demonstrating that the predictive capacity of the DCS model is largely preserved across discretisation levels within the investigated range. Moreover, we demonstrated that discretising the long polymer chains yields a pronounced reduction in computational cost, thereby improving the suitability of the DCS model for applications involving complex three-dimensional (3D) geometries (Figure 5).

Although the hybrid discretisation implementation strategy does not appear to change the global model prediction, some nuance is required. As indicated by Equation 14, polymer chain discretisation affects the expected mass loss under random chain scission. Introducing a hybrid discretisation strategy was therefore essential to prevent this artefact from influencing the degradation response. The sensitivity analysis for the hybrid discretisation threshold revealed that lowering the threshold, and thus discretising progressively shorter chains, leads to deviations in mass loss relative to the fully non-discretised reference. In contrast, the molecular weight evolution remains unaffected, consistent with earlier observations by Soares and Zunino (2010). This indicates that a discretised polymer chain implementation can influence the physical interpretation if not properly controlled. In addition to these effects observed for random chain scission, discretisation also influences the modelling of chain-end scission. While Soares et al. (2010) did not investigate how discretisation affects chain-end scission modelling, Equation 15 shows that the effective mass loss rate is directly influenced by the discretisation level. Owing to the simplicity of this dependency, the discretisation effect can be compensated for by scaling the chain scission rate according to Equation 16. As demonstrated in Figures 6C,D, this correction successfully eliminates discretisation-induced differences in both the weight-average molecular weight and mass loss evolution. Minor transition effects remain in the vicinity of the hybrid discretisation threshold. However, simulations over extended timeframes indicated that these deviations seemed to decrease over time, confirming the numerical robustness of the hybrid discretisation approach. This shows that discretisation-induced artefacts are not inherent limitations of the implementation approach, but can be corrected in a physically consistent manner, although this correction may need to be reassessed for more complex degradation mechanisms.

Although the proposed hybrid discretisation reduces the resolution with which the chain length distribution is represented, it should not be interpreted as a physically motivated coarse-graining strategy. In conventional bead-spring, rod-chain, and related polymer models, the discretisation entities typically represent physically meaningful structural units and are therefore associated with chain conformations, entanglements, chain mechanics, and other microstructural phenomena. In contrast, the DCS framework considered here tracks only the evolution of the polymer chain length distribution during degradation and does not explicitly represent chain conformations or configurational degrees of freedom. Consequently, the discretisation size is not intended to represent physical length scales such as the Kuhn length, persistence length, or entanglement length. Rather it is an implementation parameter introduced to improve computational efficiency while preserving the predictions of the underlying DCS model.

To assess the practical applicability of the discretised DCS formulation, a case study was performed in which the parameters were optimised using a GA-based approach to reproduce the *in vitro* degradation behaviour of P4MC reported by Antheunis et al. (2009) (Antheunis et al., 2009). The resulting parameters provide a close match to the experimental trends in both number- and weight-average molecular weight as well as mass loss, indicating that the DCS model implemented with a hybrid polymer chain discretisation strategy allows to accurately solve for the model’s description of the hydrolytic degradation behaviour of P4MC, an aliphatic polyester. This agreement demonstrates that the hybrid discretisation implementation strategy preserves the internal consistency of the DCS model when applied to experimental data, despite the different representation of the polymer chain. The same case study was previously performed by Pan and Brassart (2023) (Pan and Brassart, 2023), and the present results are broadly consistent with their findings. This agreement supports the internal consistency of the discretised DCS formulation despite the introduction of hybrid chain representation. A notable difference is that Pan and Brassart observed an approximately linear mass-loss evolution, whereas the current model predicts an accelerating mass loss profile, which aligns more closely with typical hydrolytic degradation behaviour of aliphatic polyesters. This difference is hypothesized to arise from the extended chain-end scission mechanism implemented in the DCS model presented here which alters the balance between chain fragmentation and solubility, thereby changing the temporal profile of mass loss.

This would enable the simulation of degradation processes in realistic 3D geometries of bioresorbable implants and allow for inclusion of diffusion of degradation products, spatial gradients, and autocatalytic effects, thereby enabling a more direct comparison with experimental observations of representative structures. This study establishes the feasibility of implementing the DCS framework using a hybrid polymer chain discretisation strategy. Importantly, the proposed implementation strategy is generic and independent of the underlying kinetic formulation. As such, the present approach can readily be extended to a full reaction-diffusion formulation and combined with more advanced DCS-based models that incorporate autocatalytic degradation mechanisms. Such an extension would enable simulations of degradation processes in realistic 3D geometries of bioresorbable implants, including diffusion of degradation products, the resulting spatial gradients, and the autocatalytic effects arising from the accumulation of acidic degradation products within the polymer bulk. In the present study, however, the analysis was intentionally restricted to a spatially homogeneous system (0D) to allow for a focused assessment of the discretised DCS implementation without the additional complexity associated with transport processes. Beyond spatial effects, the modelling approach can be further extended to account for additional physicochemical processes occurring during polymer biodegradation. As degradation progresses, increased chain mobility may lead to recrystallisation in semi-crystalline polymers. Previous studies have incorporated this effect using adapted implementations of Avrami’s theory in combination with simplified bi-modal chain scission models (Gleadall et al., 2014; Abaei et al., 2024). Including such mechanisms would enable the preferential degradation of amorphous over crystalline regions to be captured. Moreover, both molecular weight and crystallinity strongly influence the mechanical behaviour of polymers. By accounting for the evolution of both properties during degradation, the impact of biodegradation on mechanical performance could be comprehensively described. While several studies have coupled degradation models to mechanical constitutive formulations (Shirazi et al., 2016; Vieira et al., 2023; Taguti et al., 2020; Su et al., 2022), only a limited number explicitly consider the combined influence of molecular weight and crystallinity on the evolution of mechanical behaviour (Polak-Kraśna et al., 2021; Abaei et al., 2023; 2024). It is important to note that, when coupling the DCS framework to models that explicitly account for polymer morphology, chain conformations, recrystallisation, or mechanical behaviour, the implications of the discretisation strategy on physical fidelity may require further investigation.

To ensure modelling credibility as the framework is extended, these developments should be accompanied by a rigorous verification, validation, and uncertainty quantification (VVUQ) workflow. This requires access to comprehensive experimental datasets, including at minimum the evolution of molecular weight, mass loss, and crystallinity, and ideally the full molar mass distribution where available. When coupling the degradation model to a mechanical constitutive formulation, additional mechanical testing at different stages of degradation is required to characterise the evolution of key mechanical properties. Furthermore, the identifiability and uniqueness of calibrated model parameters were not explicitly assessed in this work and require further investigation, particularly when multiple model mechanisms interact.

In conclusion, this study demonstrates that computational efficiency can be improved without compromising the physical consistency of degradation modelling within the DCS framework. In particular, the results show that hybrid polymer chain discretisation, when properly designed and corrected for discretisation-induced effects, preserves the internal consistency of the model and enables accurate representation of both molecular weight evolution and mass loss. Furthermore, the modelling framework is shown to capture experimentally observed degradation trends. These findings directly address the balance between computational feasibility and model fidelity by establishing hybrid discretisation as a robust and computationally efficient pathway for advancing DCS-based degradation modelling towards more realistic and application-driven scenarios.

## Data Availability

The original contributions presented in the study are included in the article/Supplementary Material, further inquiries can be directed to the corresponding author.
