# Supplementary material for: Hybrid polymer chain discretisation for efficient and accurate implementation of hydrolytic chain scission models
Source: Front Bioeng Biotechnol. 2026 Oct 1;14:1903132. doi: 10.3389/fbioe.2026.1903132 (PMC13669098; doi:10.3389/fbioe.2026.1903132)
Supplement: Supplementary file 1 [file DataSheet1.PDF]

Supplementary Material

This Supplementary Material provides detailed derivations of the equations introduced in the Materials and Methods section of the main text. Section 1 derives Equation 5, which imposes a constraint on the coefficients k, used in the DCS model to ensure physical consistency. Subsequently, the derivations of the coefficient k are presented for three specific chain scission mechanisms: random chain scission, chain-end scission, and extended chain-end scission, presented in Sections 2, 3, and 4, respectively.

1 DERIVATION OF PHYSICAL CONSTRAINT ON COEFFICIENTS $k_{x,y}$ (EQUATION 5)

Hydrolytic chain scission is purely a bond breaking process. Physically, each scission event generates exactly one additional polymer chain (Equation 3) , while the total number of monomers remains conserved (Equation 4). These physical requirements impose a constraint on the coefficients $k_{x,y}$, which is derived below.

Starting from Equation 3 and using the definition of the dimensionless coefficient ν_x (Equation 2), the following expression is obtained:

$$\begin{aligned} 1 &= \sum_{x=1}^{N} \nu_x \\ &= \sum_{x=1}^{N} \left[-\sum_{y=1}^{x-1} k_{x,y} w_x + \sum_{y=x+1}^{N} (k_{y,x} + k_{y,y-x}) w_y \right] . \end{aligned} \tag{S1}$$

Here, ν_x is the dimensionless coefficient indicating how many chains of length x are generated or removed per scission event, and N denotes the maximum polymer chain length considered. Furthermore, $k_{x,y}$ represents the probability that a polymer chain of length x undergoes scission at bond position y, normalised with respect to the total number of chain scission events per unit volume, while w_x is the weight fraction of chains of length x.

The first term in Equation S1 can be rewritten as:

$$-\sum_{x=1}^{N} \sum_{y=1}^{x-1} k_{x,y} w_x = -\sum_{x=2}^{N} w_x \sum_{y=1}^{x-1} k_{x,y} \, . \tag{S2}$$

Reordering the summations in the second term of Equation S1 yields:

$$\sum_{x=1}^{N} \sum_{y=x+1}^{N} (k_{y,x} + k_{y,y-x}) w_y = \sum_{y=2}^{N} w_y \sum_{x=1}^{y-1} (k_{y,x} + k_{y,y-x}) \, . \tag{S3}$$

Substituting Equations S2 and S3 back into Equation S1 and renaming the summation indices in the second term gives:

$$\begin{aligned}\sum_{x=1}^{N} \nu_x &= -\sum_{x=2}^{N} w_x \sum_{y=1}^{x-1} k_{x,y} + \sum_{y=2}^{N} w_y \sum_{x=1}^{y-1} (k_{y,x} + k_{y,y-x}) \\ &= -\sum_{x=2}^{N} w_x \sum_{y=1}^{x-1} k_{x,y} + \sum_{x=2}^{N} w_x \sum_{y=1}^{x-1} (k_{x,y} + k_{x,x-y}) \,.\end{aligned} \tag{S4}$$

Using the index substitution $z = x - y$, the second term can be rewritten as shown in Equation S5. Applying this substitution to Equation S4 leads to Equation S6.

$$\sum_{y=1}^{x-1} k_{x,x-y} = \sum_{z=1}^{x-1} k_{x,z} \tag{S5}$$

$$\begin{aligned}\sum_{x=1}^{N} \nu_x &= -\sum_{x=2}^{N} w_x \sum_{y=1}^{x-1} k_{x,y} + 2\sum_{x=2}^{N} w_x \sum_{y=1}^{x-1} k_{x,y} \\ &= \sum_{x=2}^{N} w_x \sum_{y=1}^{x-1} k_{x,y} = 1\end{aligned} \tag{S6}$$

Equation S6 is identical to Equation 5 presented in the Materials and Methods section of the main text, and establishes the physical constraint on the coefficients $k_{x,y}$.

2 DERIVATION OF COEFFICIENT K FOR RANDOM CHAIN SCISSION (EQUATION 6)

Starting from the normalisation constraint on the coefficients $k_{x,y}$ derived in Section 1, analytical expressions for the coefficients can be derived for different chain scission mechanisms. Random chain scission is characterised by an equal probability of hydrolytic bond cleavage at every position along the polymer chain. Consequently, the scission coefficients are independent of chain length and bond position, such that $k_{x,y} = k$, regardless of the value of x or y. Substituting this definition into the constraint imposed on the coefficients $k_{x,y}$ (Equation 5) gives:

$$\begin{aligned}1 &= \sum_{x=2}^{N} w_x \sum_{y=1}^{x-1} k_{x,y} \\ &= \sum_{x=2}^{N} w_x \sum_{y=1}^{x-1} k \,.\end{aligned} \tag{S7}$$

Since the coefficient k is independent of bond position and chain length in the case of random chain scission, the second summation can be further simplified as:

$$\begin{aligned} 1 &= \sum_{x=2}^{N} w_x(x-1)k \\ &= \sum_{x=1}^{N} w_x(x-1)k \,. \end{aligned} \tag{S8}$$

By definition, the total concentration of bonds is given by $C_b = \sum_{x=1}^{N} w_x(x-1)$. Substituting this relation into Equation S7 yields the following expression for the coefficient k associated with random chain scission:

$$k = \frac{1}{\sum_{x=1}^{N} w_x(x-1)} = \frac{1}{C_b} \,. \tag{S9}$$

Equation S9 is identical to Equation 6 presented in the Materials and Methods section of the main text. Equation S9 therefore defines the normalised scission coefficient associated with a purely random chain scission mechanism.

3 DERIVATION OF COEFFICIENT K FOR CHAIN-END SCISSION (EQUATION 8)

Using the normalisation constraint on the coefficients $k_{x,y}$ derived in Section 1, the coefficient k can similarly be derived for the specific case of chain-end scission. In the case of chain-end scission, only the outermost bond of a polymer chain is susceptible to hydrolysis, as mathematically defined in Equation 7 of the Materials and Methods section in the main text. Consequently, only terminal bonds contribute to the scission process. Applying this chain-end scission definition to the physical constraint given in Equation 5 results in:

$$\begin{aligned} 1 &= \sum_{x=2}^{N} w_x \sum_{y=1}^{x-1} k_{x,y} \\ &= w_2 k + \sum_{x=3}^{N} w_x(2k) \,. \end{aligned} \tag{S10}$$

In this equation, chains consisting of two monomer units (with weight fraction w_2) are treated separately because they contain only a single bond between neighbouring monomers. In contrast, chains with length $x \geq 3$ contain two susceptible terminal bonds, each contributing equally to the scission probability. Additionally, this expression can be rearranged as follows:

$$\begin{aligned} 1 &= k \left(w_2 + 2 \sum_{x=3}^{N} w_x \right) \\ &= k \left(-2w_1 - w_2 + 2 \sum_{x=1}^{N} w_x \right) \,. \end{aligned} \tag{S11}$$

By definition, the total polymer chain concentration is given by $W_p = \sum_{x=1}^{N} w_x$. Substituting this definition into Equation S11 gives the following expression for the coefficient k associated with chain-end scission:

$$
\begin{aligned}
k &= \frac{1}{\left(2\sum_{x=1}^{N} w_x - w_2 - 2w_1\right)} \\
&= \frac{1}{(2W_p - w_2 - 2w_1)} \,.
\end{aligned}
\tag{S12}
$$

Equation S12 is therefore identical to Equation 8 presented in the Materials and Methods section of the main text, and defines the normalised scission coefficient associated with chain-end scission.

4 DERIVATION OF COEFFICIENT K FOR EXTENDED CHAIN-END SCISSION (EQUATION 10)

In extended chain-end scission, the a terminal bonds at both ends of the polymer chain are susceptible to hydrolysis, as mathematically defined in Equation 9 in the Materials and Methods section of the main text. Similar to the derivation presented for chain-end scission, the coefficient k can be obtained from the physical constraint defined in Equation 5 as follows:

$$
\begin{aligned}
1 &= \sum_{x=2}^{N} w_x \sum_{y=1}^{x-1} k_{x,y} \\
&= \sum_{x=2}^{2a} w_x (x-1)k + \sum_{x=2a+1}^{N} w_x(2ak) \,.
\end{aligned}
\tag{S13}
$$

Chains with length $x \leq 2a$ contain fewer than $2a$ susceptible terminal bonds and therefore contribute proportionally to their chain length. This behaviour is captured in the first term of Equation S13. In contrast, longer chains contribute with the maximum possible number of susceptible bonds, equal to $2a$. Equation S13 can be further rearranged as follows:

$$
\begin{aligned}
1 &= \sum_{x=2}^{2a} w_x \left(-2a + (x-1)\right) k + \sum_{x=1}^{N} w_x(2ak) \\
&= k\left[-\sum_{x=1}^{2a} \left(2a - (x-1)\right) w_x + 2a\sum_{x=1}^{N} w_x\right] \,.
\end{aligned}
\tag{S14}
$$

By definition, the total polymer chain concentration is given by $W_p = \sum_{x=1}^{N} w_x$. Substituting this relation into Equation S14 yields the following expression for the coefficient k associated with extended chain-end scission:

$$
\begin{aligned}
k &= \frac{1}{2a\sum_{x=1}^{N} w_x - \sum_{x=1}^{2a} \left(2a - (x-1)\right) w_x} \\
&= \frac{1}{2aW_p - \sum_{x=1}^{2a} \left(2a - (x-1)\right) w_x} \,.
\end{aligned}
\tag{S15}
$$

Equation S15 is therefore identical to Equation 10 presented in the Materials and Methods section of the main text, and defines the normalised scission coefficient associated with extended chain-end scission.
